# Editorial: MAIT cells come of age

**DOI:** 10.3389/fimmu.2023.1281881

**Published:** 2023-09-06

**Authors:** Alexandra J. Corbett, James E. Ussher, Timothy S. C. Hinks

**Affiliations:** ^1^ Department of Microbiology and Immunology, Peter Doherty Institute for Infection and Immunity, University of Melbourne, Melbourne, Australia; ^2^ Department of Microbiology and Immunology, University of Otago, Dunedin, New Zealand; ^3^ Respiratory Medicine Unit and National Institute for Health Research Oxford Biomedical Research Centre, John Radcliffe Hospital, Oxford, United Kingdom

**Keywords:** MAIT cells, metabolite antigens, MR1, microbiome, viral infection, mouse models, immune cell interaction

Mucosal-associated Invariant T (MAIT) cells recognise microbial metabolite-derived antigens presented by the MHC Class I related (MR1) protein (1, 2). In the decade since MR1-MAIT antigen discovery, the role of MAIT cells in anti-microbial immunity has been well defined, with diverse functions, including maintenance of barrier integrity and tissue repair, being described more recently. MAIT cells can drive pathology in chronic infections and have been associated with poor outcomes in certain autoimmune diseases, cancer, and COVID-19 (3–5). Current research considers the impact of host genetics, interactions of MAIT cells with pathogens, other immune cells, stromal cells, and the microbiome, and looks towards therapeutic targeting of this unconventional immune axis.

Humans with natural immune cell deficiencies provide unique insight into the relevance of cell subsets in health. MAIT cell deficiencies were initially elusive, but as the field has developed and their frequencies are now routinely assessed in clinical immunology, MAIT deficiency and dysfunction has been described in several studies. Howson and Bryant summarise these, allowing common themes to emerge. MAIT cells are reduced in a wide range of inborn errors of immunity that are associated with bacterial, fungal or viral infections. However, as these occur as part of wider immune disruptions, it is hard to attribute causality. Interestingly in rare cases of pure MAIT deficiency, a tendency to viral infections was as prominent a feature as bacterial infections, underlining the importance of both TCR-dependent and TCR-independent functions.

MAIT cell activation during viral infections is mediated via cytokines and independent of MR1 (6). Intriguingly, viral downregulation of MR1 expression, reminiscent of immune evasion mechanisms impacting MHC Class I antigen presentation, has been described (7, 8). Ashley et al. investigate the ability of human cytomegalovirus (HCMV), a betaherpesvirus known to cause immune dysregulation, to dampen MR1-MAIT immunity. They show HCMV can suppress MR1 expression in infected fibroblasts, resulting in the inhibition of MAIT cell activation. As previously reported for MHC Class I, bystander cells increased their expression of MR1. The authors identify HCMV glycoprotein gpUS9 as an immune evasion molecule capable of targeting MR1. Its impact on TCR-dependent MAIT cell activation by concomitant bacterial infections remains to be determined.

Varicella Zoster Virus (VZV) can similarly impact MR1 expression (8). Here, Purohit et al. demonstrate another VZV-MAIT cell interaction; the direct productive infection of MAIT cells by VZV, with infected cells showing higher expression of activation and proliferation markers, and higher frequencies of infection in the CD4-expressing subsets. Their results, including retained expression of migration and skin-homing markers in infected cells, and the ability to transmit virus to other cells, raise the possibility that MAIT cells may act as a Trojan horse, contributing to viral dissemination throughout the host.

MAIT cells can produce IFN-γ following stimulation (9–11), but while they express receptors for and are responsive to type I interferons (6, 12), their role in producing type I interferon is less appreciated. Jakob et al. assessed the MAIT cell translatome, using CLICK chemistry to identify newly synthesized proteins from MAIT cells in response to antigen. Consistent with single-cell transcriptomic data, 5-(2-oxopropylideneamino)-6-D-ribitylaminouracil (5-OP-RU) induced MAIT cell production of several type I IFN signalling proteins. The signals driving this response are yet to be determined. This study also showed evidence that MR1-activated MAIT cells could promote M1 macrophage polarisation, highlighting the role MAIT cells may play in influencing monocyte and macrophage phenotype, with potential consequences for immunity during pathogen responses and homeostasis.

While the MR1-MAIT axis is highly conserved, polymorphisms in other immune genes may impact MAIT immunity. MAIT cells have high IL7R expression and IL-7 is important for their homeostasis and activation. MAIT cells are depleted and functionally exhausted in a range of viral infections, including HIV (13–16). As IL-7 correlates with MAIT cell frequencies in HIV infected individuals, Han et al. studied the impact of IL7RA polymorphisms on circulating MAIT cells in healthy individuals and HIV infected individuals after long-term anti-retroviral therapy (ART). While MAIT cell dysfunction in chronic HIV was not associated with IL17RA haplotype, ‘haplotype 2’ was associated with increased MAIT numbers in healthy controls and greater recovery following ART.

While T cell metabolic changes are essential for effector function, MAIT and other innate-like T cells sit outside the norms of naïve, activated and effector definitions as they exit the thymus in an effector-memory-like state (17, 18). Several studies have assessed MAIT cells in the context of metabolic diseases, but the metabolism of MAIT cells themselves is just emerging. Kedia-Mehta and Hogan review current knowledge both on their functions in metabolic disease and how alterations in metabolism impact MAIT immunity.

The MR1-MAIT axis is both stimulated by microbial antigens from the commensal microbiome and contributes to the shaping of the microbiome. As MAIT cells become more widely studied, and as the analytic power of microbial metagenomics increases, we are discovering more about these interactions. A review by Jabeen and Hinks integrates findings and speculates on future therapeutic manipulation of MAIT cells by antigens or microbiota. It will be important to understand the functions of MAIT cells during homeostasis, which may yet prove to be their most influential role.

MAIT cell anti-bacterial protective responses have been demonstrated in mouse studies with bacterial pathogens that cause marked expansion of MAIT cells, while the low frequency of MAIT cells in laboratory mice relative to humans makes their assessment difficult in other settings. Building on previous methods of expanding MAIT cells by the same group (19–21), Nelson et al. demonstrate the utility of the MAIT antigen precursor 5-amino-6-D-ribitylaminouracil (5-A-RU) in expansion and activation protocols to enable further MAIT cell study in mice, and also speculate on 5-A-RU as a common precursor of diverse MAIT antigens.

Overall, this Research Topic points towards a bright future as the field considers therapy informed by the specific functions of MAIT cells and their role in different immune contexts. As MAIT cells come of age, *in vivo* studies and clinical trials of MAIT cell therapies will be the next frontier for this intriguing T cell subset.

## Author contributions

AC: Conceptualization, Writing – original draft, Writing – review & editing. JU: Conceptualization, Writing – original draft, Writing – review & editing. TH: Conceptualization, Writing – original draft, Writing – review & editing.
